# Supplementary material for: From Epimedium to Neuroprotection: Exploring the Potential of Wushanicaritin
Source: Foods. 2024 May 11;13(10):1493. doi: 10.3390/foods13101493 (PMC11119816; doi:10.3390/foods13101493)
Supplement: Supplementary file 1 [file foods-13-01493-s001.zip › foods-2980602-supplementary.pdf]

Table S1. The primer sequence used in the present study.

Gene	Primer sequence	Pruduct size (bp)
SOD1	Forward: GCCGTGTGCGTGCTGAAGG<br>Reverse: ACAACTGGTTCACCGCTTGCC	80
SOD2	Forward: GCTGGAGGCTATCAAGCGTGAC<br>Reverse: TTAGAGCAGGCGGCAATCTGTAAG	147
GPx1	Forward: AGGTCCAGACGGTGTTCCAGTG<br>Reverse: TAGGGGTTGCTAGGCTGCTTGG	105
CAT	Forward: CGCCTGGGACCAAACTATCTGC<br>Reverse: TCTGGTGCGCTGAAGCTGTTG	146
Bcl-2	Forward: GCCTGAGAGCAACCGAACGC<br>Reverse: AGGTGGCACAGGGCTGAGC	109
Bax	Forward: CCAGGACGCATCCACCAAGAAG<br>Reverse: GCTGCCACACGGAAGAAGACC	138
Caspase-3	Forward: TTTGGAACGAACGGACCTGTGG<br>Reverse: ACCGCAGTCCAGCTCTGTACC	132
Caspase-7	Forward: TCCTGCTGAGCCACGGAGAAG<br>Reverse: CGGCACGCCTGGATGAAGAAG	142

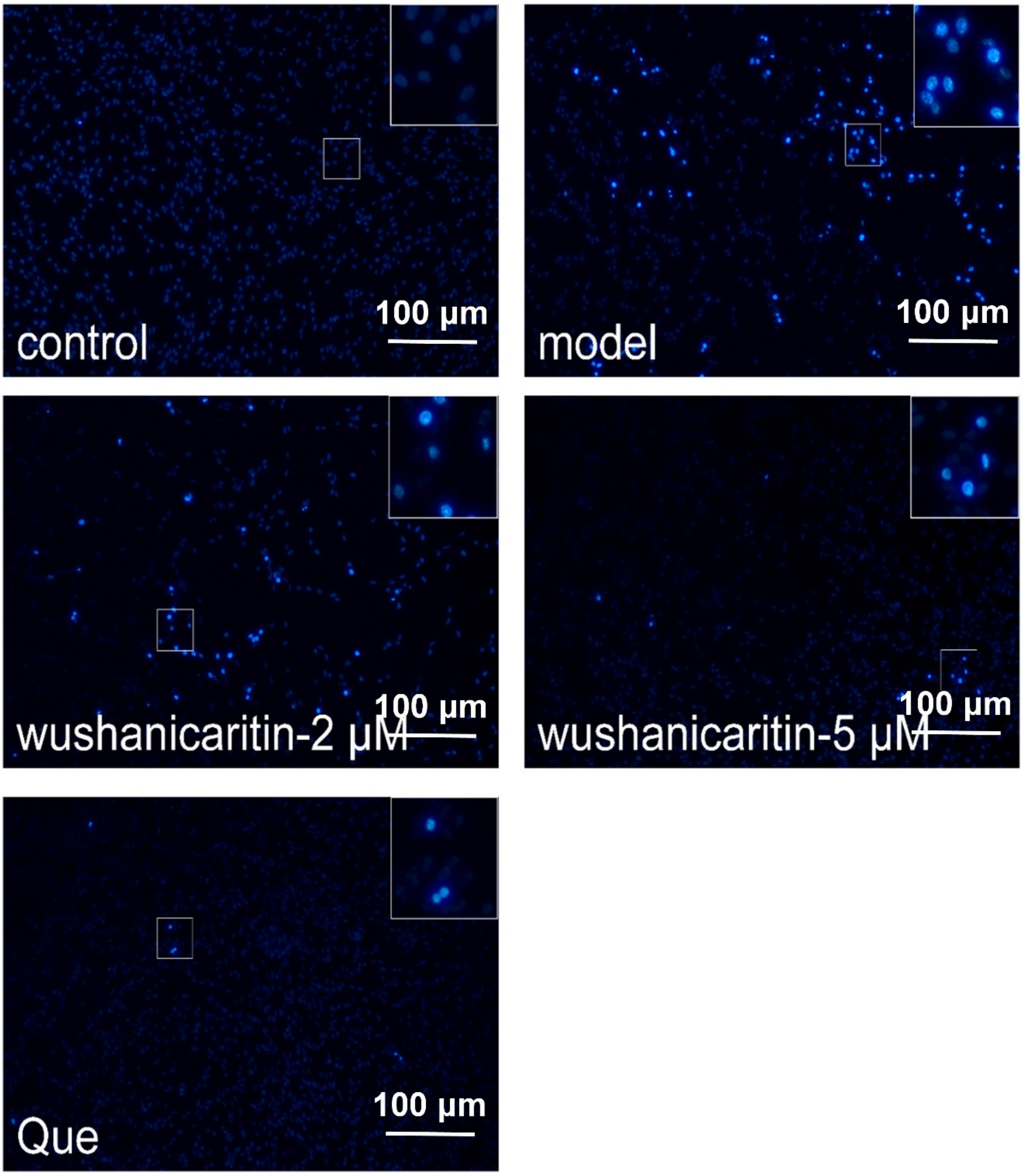

Figure S1. Effects of wushanicaritin and quercetin on the morphology of nuclear chromatinsin PC12 cells. Control group refers to PC-12 cells with vehicle treatment (0.1% DMSO); Model group refers to PC-12 cells treated with glutamate; wushanicaritin-2 μM, wushanicaritin-5 μM and Que refers to PC-12 cells co-treated with glutamate and wushanicaritin at concentrations of 2, 5 μM, or quercetin (30 μM), respectively. Scale bar=100 μm.
